# Open approaches for cruciate ligament reconstruction in knee dislocations: A technical note and case series

**DOI:** 10.1051/sicotj/2021016

**Published:** 2021-03-22

**Authors:** Michael Held, Martiz Laubscher, Richard von Bormann, Dustin L. Richter, Daniel C. Wascher, Robert C. Schenck

**Affiliations:** 1 Department of Orthopaedic Surgery, Groote Schuur Hospital, Orthopaedic Research Unit, University of Cape Town 7925 Cape Town South Africa; 2 Knee Unit, Groote Schuur Hospital and Christiaan Barnard Memorial Hospital, University of Cape Town 7700 Cape Town South Africa; 3 Department of Orthopaedics & Rehabilitation, The University of New Mexico Health Sciences Center Albuquerque 87131-0001 NM USA

**Keywords:** Knee dislocation, Multi-ligament knee injury, Multiple ligamentous injuries, Open approach, Limited resource settings (LRS)

## Abstract

*Introduction*: Arthroscopic surgery is the gold standard for cruciate ligament reconstruction in multi-ligament knee injuries. However, hospitals in limited-resource settings often lack arthroscopic-trained surgeons or equipment. Open approaches for treating knee dislocations can overcome many of these limitations. *Methodology*: This study aims to describe techniques for open approaches in a supine patient to address the cruciate ligaments in multi-ligament knee injuries and to review associated complications and clinical outcomes in a retrospective case series. *Results*: Ten patients with multi-ligament knee injuries who had undergone open cruciate ligament reconstruction between July 2016 and November 2018 were retrospectively identified. Open approaches were performed owing to the extravasation of arthroscopy fluid into the posterior compartment (3) or a large traumatic arthrotomy (7). Complications and patient-reported outcomes were analysed. Eight of the 10 patients were followed up at 10 months postoperatively (range, 5–23 months). None had iatrogenic neurovascular damage. Median outcomes scores were: visual analogue scale, 45 (range, 0–100); Knee Injury and Osteoarthritis Outcome Score-Physical Function Short Form, 81.4 (range, 75–100); Lysholm, 85 (range, 67–92). *Discussion*: Open approaches were safe and useful in treating cruciate ligaments and should be considered in arthroscopy fluid extraversion and large traumatic arthrotomies.

## Introduction

For multi-ligament knee injuries (MLKIs), most authors promote an arthroscopic reconstruction of cruciate ligaments and open surgical treatment of lateral and medial structures to achieve good outcomes [1–4]. Arthroscopy can also help to assess and treat associated meniscal and cartilaginous injuries, decrease the risk of arthrofibrosis, and result in less injury to the articular cartilage.

**Figure 1:**
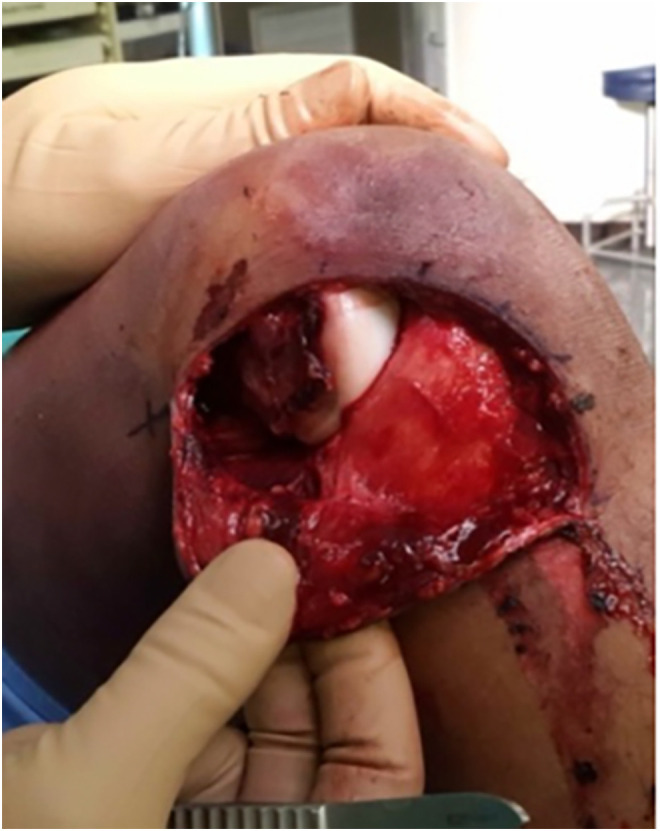
Large medial capsular tear with exposed medial condyle after skin incision in an irreducible knee dislocation. This allows access to the tibial insertion of the posterior cruciate ligament through the traumatic arthrotomy without extensive surgical dissection.

But the risk of arthroscopy, especially in acute MLKIs, is fluid extravasation and the concomitant risk of compartment syndrome or vascular compromise. Also, in knee dislocations with a large Morel-type lesion in which traumatic dissection can be used to access ligaments (Figure 1), arthroscopy is potentially unnecessary. In some circumstances, such as an irreducible knee dislocation, an open approach is the only safe initial approach [5–7]. Thus, even surgeons well versed in arthroscopic techniques need to be familiar with alternatives such as open approaches for cruciate ligament reconstruction. Open approaches for treating knee dislocations can overcome many of these limitations and allow surgeons to stabilize the knee without the need for specialized arthroscopic equipment or skills.

There is a paucity of studies on open cruciate reconstruction from state hospitals in a limited resource setting (LRS). Yet, these centres often lack arthroscopic-trained surgeons and equipment for which open techniques play a vital role to provide trauma care for a large patient population [8]. Also, a comprehensive description of open access to cruciate ligaments through various approaches in a single publication is not available.

The purpose of this study was to present open approaches used to address both cruciate ligaments in patients with MLKIs. Our aims were as follows: (1) describe surgical approaches that can be useful to treat patients with acute injuries and in hospitals in LRS, and (2) report short-term clinical outcomes of ten patients with knee dislocations treated with open cruciate ligament reconstruction by a single surgeon (MH) in an LRS.

## Methods

The primary aim of this work is to provide a technical note for open approaches in a supine patient to address the cruciate ligaments in multi-ligament knee injuries. Furthermore, a case series of patients treated with these techniques were reviewed retrospectively to describe associated complications and clinical outcomes.

### Surgical technique

Patients are positioned supine to allow full knee flexion and extension. A radiolucent table enables fluoroscopy if necessary. A foot bolsters placed under the heel keeps the knee flexed 70°, allowing easier access to the tibial footprint of the anterior cruciate ligament (ACL). With the toes on the bolster, knee flexion can be increased to allow easier access to the femoral insertion of the cruciate ligaments. A high side bolster prevents the leg from rotating at the hip. Tourniquets, when used, are placed as proximal as possible to allow insertion of guidewires without compromise.

#### Incision

The primary incision is curvilinear anteromedial beginning halfway between the medial border of the patella and the medial epicondyle, extending distally along the anteromedial surface of the tibia to below the pes anserine attachment. This incision allows access to the intercondylar notch, the proximal and distal attachments of the medial collateral ligament, and the tibial insertion of the PCL (i.e., Lobenhoffer approach, Video 1). It also allows harvesting of the patella tendon and hamstring tendons and could be extended proximally for a quadriceps tendon harvest. For lateral side injuries, the lateral incision begins over the lateral epicondyle and extended distally posterior and inferior to the fibular head. This incision gives access to the peroneal nerve, the posterolateral structures, and the PCL tibial attachment. It also facilitates outside-in drilling and fixation of the ACL femoral attachment.

The decision of whether to approach the posterior part of the knee laterally or medially is dictated by the collateral ligament involvement. This is usually confirmed via magnetic resonance imaging or stress radiographs. The approach to the PCL through the medial Lobenhoffer interval is facilitated by traumatic dissection of posteromedial structures and capsules. Access through the Lobenhoffer interval is performed by the takedown of the semimembranosus muscle as needed, and then using the plane between the medial collateral ligament (MCL) and medial head of the gastrocnemius with an elevation of the popliteus muscle. Similarly, posterolateral corner (PLC) injuries facilitated the approach to the PCL through a lateral incision [9].

#### Notch access

**Figure 2:**
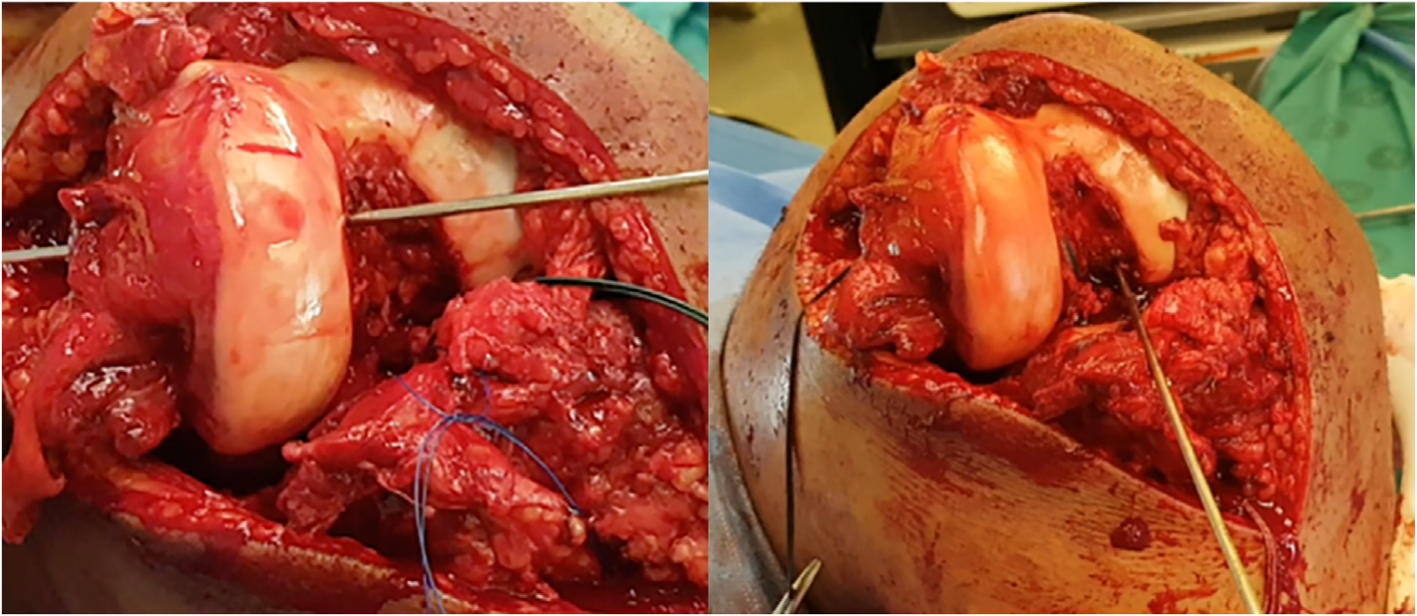
Intraoperative access to the notch of left through a medial parapatellar dissection with the patella retracted. This gives appropriate access to important structures in the notch. *Left*: guidewire drilled through the femoral footprint of the anterolateral bundle of the posterior cruciate ligament. *Right*: guidewire drilled through the femoral footprint of the anterior cruciate ligament.

Access to the notch is achieved through a medial parapatellar dissection (Figure 2, Video 2). This can be extended subvastus, midvastus, or proximally into the quadriceps tendon to enable patella sublaxation, greatly increasing exposure [10]. Although the fat pad may be retained [11, 12], it is frequently debulked to increase visualization. The cruciate ligament stumps are excised to allow better visualization of the insertion sites for guide pin placement. The knee is placed in slight extension to enable visualization of the tibial insertion of the ACL, as well as the anterior horns and roots of the menisci. It also facilitated the placement of the ACL tibial and PCL femoral tunnels by removing tension off the extensor mechanism. If cruciate ligament guides are available, the use of Z-retractors and fat pad excision can provide acceptable visualization of the notch even in more limited incisions. A headlamp can provide clearer visualization.

Guide pins for the femoral attachments for the ACL and PCL are placed in an “inside-out” fashion. When performing a single bundle PCL reconstruction, the guide pin is started in the centre of the anterolateral bundle. The ACL pin is started at the centre of the ACL femoral footprint. The ACL tibial guide pin is placed in an “outside-in” fashion.

#### Medial approach

The posterior aspect of the knee can be approached through the Lobenhoffer interval [9, 13]. The superficial dissection should expose the hamstrings and MCL. To increase access, the knee was flexed to 90° with the ipsilateral hip externally rotated. Visualization is improved by working from the opposite side of the table. A sponge can be used to clean residual fat off these structures to identify the proximal and distal border of the pes anserine.

For acute injuries to the posteromedial corner, the traumatic dissection often enables palpation of the champagne glass drop-off and PCL stump around the posteromedial part of the knee. To gain further access, a Hohman retractor is placed anterior to the medial head of the gastrocnemius muscle.

**Figure 3:**
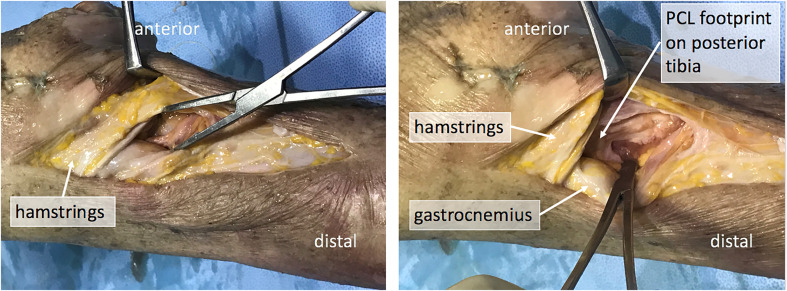
Medial Lobenhoffer approach in a left cadaveric knee. *Left*: the haemostat shows access to the posterior part of the knee, inferior to the attachments of the hamstrings. *Right*: the gastrocnemius muscle is reflected posteriorly to give access to the posterior capsule and posterior cruciate ligament. Note the haemostat is spreading fibres of the popliteus muscle. Leaving the pes intact and working above and below the pes in knee flexion allows for safe access to the back of the tibia.

External rotation of the tibia and blunt dissection of the fibres of the popliteus muscle gives access to the posterior capsule and PCL tibial insertion (Figure 3, Video 3). Working with a blunt instrument, such as a Cobb (Skylar Surgical Instruments, West Chester, Pennsylvania), the residual capsule was stripped until full access and visualization of the PCL insertion is possible. It is critical to keep the retractors anterior to the gastrocnemius and popliteal muscles, hugging the posterior surface of the tibia to protect the neurovascular structures during tibial tunnel drilling or through creation. Knee flexion to 60–90° during dissection of the back of the tibia relaxes the neurovascular structures. Adhesions and scarring in chronic injuries can make the anatomic differentiation of structures challenging.

Use of a Cobb elevator, electrocautery, identifications of anatomic landmarks of the medial joint line and the inner edge of the posteromedial femoral condyle, and exposure through the Lobenhoffer interval allow improved visualization and safe access to the posterior knee in these multiple trauma patients. This helps avoid prone positioning and subsequent physiologic risk to already compromised patients.

#### Lateral approach

**Figure 4:**
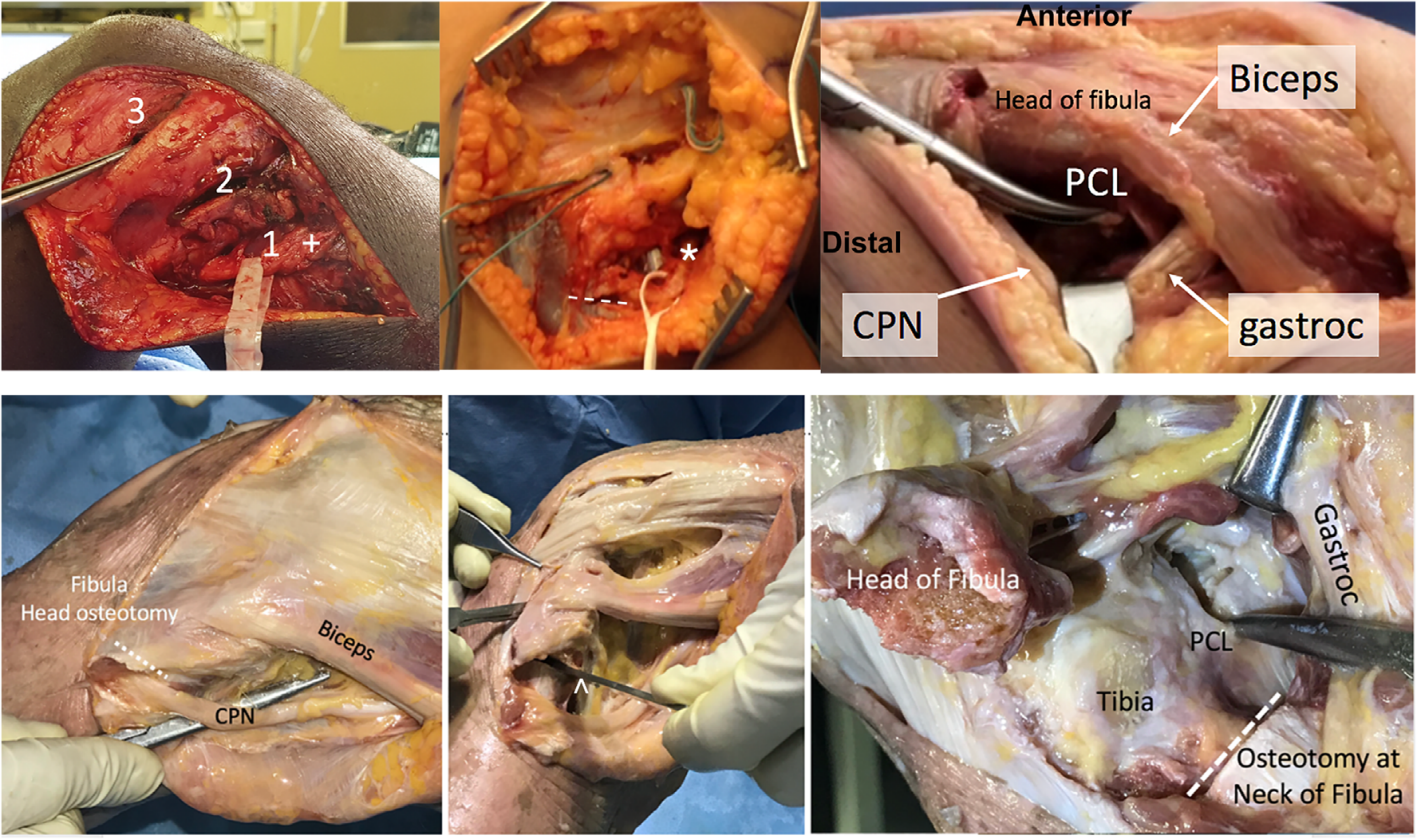
Lateral approach to careful dissection and retraction of common peroneal nerve (CPN). *Top left*: release of the nerve through window “2”, in which the nerve has (+) a drop-down appearance with appropriate release below window “1” and the forceps are placed into window “3”. *Top middle*: release of peroneal nerve (*) in a chronic condition with extensive scarring and release to peroneus longus fascia (white line). *Top right*: lateral Lobenhoffer approach without fibula head osteotomy in a cadaver, in which protecting the CPN can be achieved through access distal to the biceps tendon, retracting the gastrocnemius (“gastroc”) tendon posterior. The tibial insertion of the posterior cruciate ligament (“PCL”) can be reached posterior to the head of the fibula. *Bottom left*: lateral Lobenhoffer approach to the posterior part of the tibia in a cadaver. Initially, the CPN is identified and protected. *Bottom middle and right*: An osteotomy at the neck of the fibula (^) increases access to the PCL when the gastrocnemius and popliteus muscles are retracted posteriorly.

Using a direct lateral incision, access to the posterior aspect of the tibia and insertion of the PCL is achieved (Figure 4, Video 4). This has been described for the reconstruction of the PLC [14–17]. The incision passes just anterior to the lateral condyle and distally just posterior to the fibular head. With a separate anteromedial incision to access the notch, a sufficient skin bridge of at least 8cm is maintained to avoid skin necrosis. Three fascial “windows” are created to gain access to the posterolateral structures [17].

An incision is first made posterior to the biceps tendon (window 1) to identify the peroneal nerve about 1cm distal and posterior to the tip of the fibular head or just distal around the fibular neck. The nerve is released distally into the anterior compartment with the careful division of the overlying muscle fascia to achieve a tension-free “drop-down sign” of the nerve. Careful dissection and protection of the nerve throughout the procedure are crucial.

Window 2 is created next by developing the interval between the iliotibial band and the biceps femoris tendon. This window gave access to the posterolateral capsule, the lateral meniscus, the fibular collateral ligament (FCL) attachment, and the attachment of the popliteofibular ligament.

Finally, window 3 is created by splitting the iliotibial band fibres overlying the lateral epicondyle. This allowed visualization of the femoral attachments of the FCL and popliteus tendon.

The posterior tibia is accessed through the lateral approach [9]. Window 1 or 2 is used to gain access posterior to the fibular head. The lateral head of the gastrocnemius is identified and retracted posteriorly with a Hohman retractor by sliding under the popliteus tendon beneath the lateral head of the gastrocnemius muscle. Blunt dissection is used to avoid venous bleeders. A fibular neck osteotomy can be used at the metaphyseal–diaphyseal junction to increase access [9]. However, with traumatic tears of the lateral collateral ligament and popliteus tendon, the exposed tibia can often be palpated around the fibular head and osteotomy is usually not necessary [18].

The tibial insertion of the PCL can be exposed by internal rotation of the tibia and placing a Hohmann retractor past the midline of the tibia, just proximal to the champagne glass drop-off. Careful posterior retraction and knee flexion to 90° will protect neurovascular structures and enable appropriate access and visualization.

#### Closure

Medially and laterally, the interval of the posterior approach is mainly intermuscular, and no deep sutures are necessary for closure. On the lateral side, window 1 should be allowed to heal without repair to avoid any compression or injury to the peroneal nerve. The interval between the biceps femoris and iliotibial band can be reapproximated with an absorbable suture. The window 3 splits of the iliotibial band should be closed with strong absorbable braided sutures.

Medially, no suture of deep structures is necessary [9]. The popliteus muscle is split in line with the fibres during the medial approach and does not require approximation. Postoperatively, it is critical to monitor limb perfusion and peripheral nerve function. Lastly, tourniquet time must be monitored, and continuous ischemia longer than 120min should be avoided. It is often difficult to perform bicruciate and lateral collateral ligament reconstructions in less than 2h. Surgeons should either allow reperfusion for at least 15min followed by tourniquet re-inflation or perform the remainder of the procedure (or the entire operation) without tourniquet control.

### Case series

For this case series, consecutive patients who had undergone surgery for MLKI between July 2016 and November 2018 were retrospectively identified. All patients who had an open cruciate reconstruction were included. Patients below the age of 18 years were excluded. Demographic data, injury mechanism, and classification, associated injuries, time delay to surgery, as well as the indication for open surgery were collected.

The primary outcome measure was the presence of any major complications such as neurovascular injury, stiffness, delayed wound healing, or infection. Secondary patient-reported outcome measures (PROMs) included the following: the visual analogue scale (VAS), Knee Injury and Osteoarthritis Outcome Score-Physical Function Short Form (KOOS-PS), and Lysholm scale.

All procedures performed in studies involving human participants were in accordance with the ethical standards of the institutional and/or national research committee (HREC REF 050/2018) and with the 1964 Helsinki declaration and its later amendments or comparable ethical standards.

## Results

Ten patients (two female) with a mean age of 35 years (range: 18–64, IQR: 21.5) were identified. All injuries, except one (ultra-low energy fall), were high-energy injuries caused by road traffic collisions. Injuries were further categorized by the Schenck classification system. Eight patients had a KDIII or higher injury and most had associated fractures or soft tissue and neurovascular injuries (Table 1). All procedures but one were performed within 3 months of injury.

**Table 1:** Categorization of knee dislocations according to Schenck classification, and description of associated injuries.

Patient no.	Sex	Age (years)	KDC	Associated injuries	Associated IA pathology
1	Male	38	KDIIIM	–	Medial meniscus bucket handle tear
2	Female	37	KDIV	–	Medial and lateral meniscus posterior root avulsion, lateral meniscus tear bucket handle
3	Male	59	KDI	–	–
4	Male	31	KDIV	–	–
5	Male	64	KDIIIL	Femur fracture, closed head injury, CPN	Arcuate fracture
6	Male	58	KDV	Open book pelvic injury, ipsilateral foot fracture, contralateral MLKI, CPN	Patella tendon rupture
7	Male	17	KDII	–	–
8	Male	32	KDIIIM	–	–
9	Female	33	KDIIIL	CPN	–
10	Male	25	KDIIIM	Contralateral MLKI with CPN, traumatic aortic dissection	Medial meniscus anterior root avulsion

Abbreviations: KDC, knee dislocation classification; IA, intraarticular; CPN, common peroneal nerve; MLKI, multi-ligament knee injury; CPNP, common peroneal nerve palsy; –, not applicable.

Three patients were converted to open surgical treatment owing to fluid extravasation during diagnostic arthroscopy. All of these patients underwent a posteromedial approach to the posterior cruciate ligament (PCL) tibial insertion. In seven patients, a large traumatic arthrotomy enabled sufficient access to the posterior tibia through the medially based injuries (five patients) and laterally based injuries (two patients). Two patients had bilateral MLKIs, of which only one side was treated through an open approach.

Eight of the 10 patients were able to be contacted for a median follow-up of 24 months (range, 17–33 months; IQR, 20.5). No iatrogenic damage to neurovascular structures occurred.

**Figure 5:**
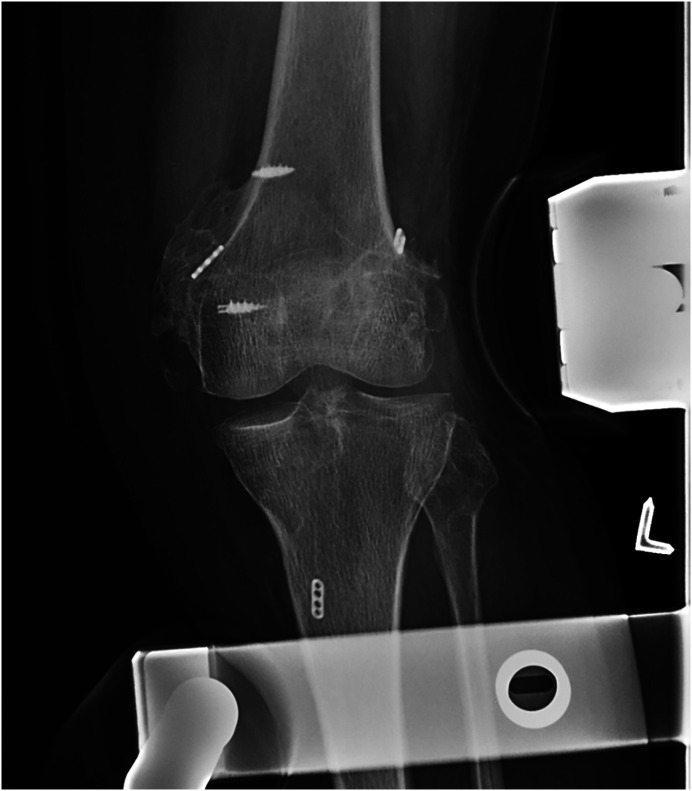
Radiographic valgus stress view of a patient with a KDIIIM, showing heterotopic ossification around the medial epicondyle at 9 months postoperatively.

Three patients developed arthrofibrosis with decreased flexion of less than 80°. Two of these patients showed heterotopic ossification on radiographs (Figure 5). One of these patients improved after manipulation under anaesthesia to 100°, whereas the other two patients opted not to have a further intervention. In one patient with a KDV and ipsilateral patella tendon rupture, impaired wound healing necessitated reoperation after 6 weeks, which resolved after removal of the protective cerclage wire.

PROMs were assessed at the time of final follow-up. The KOOS-PS median score was 81.4 (range, 75–100; IQR, 12.2) and the median Lysholm score was 85 (range, 67–92; IQR, 13.3). The median VAS pain rating was 45 (range, 0–50; IQR 15).

## Discussion

Arthroscopic single-stage surgery is the gold-standard for cruciate reconstruction in multi-ligament knee injuries. But, open cruciate surgery avoids fluid extravasation and can overcome challenges faced by the hospital with limited arthroscopy-trained surgeons or equipment in an LRS (Table 2). The findings of this study show that open cruciate surgery in 10 patients with severe MLKIs resulted in acceptable PROMs, with an acceptable incidence of complications. Notably, arthrofibrosis and heterotopic ossification (HO) occurred in three patients.

**Table 2:** Key points for open cruciate procedures.

No.	Description
1	Use traumatic dissection of posterolateral and posteromedial corners to gain access to the PCL insertion of the tibia
2	Minimize tourniquet use to prevent prolonged ischemia
3	For the retraction of posterior structures, stay subperiosteal and dissect past the midline prior to placing the Hohmann retractor
4	Even a small shift or subluxation of the patella out of the notch increases access and visualization of the cruciate ligaments greatly
5	Decreasing flexion of the knee allows better access to the notch
6	Sit during the dissection of the posterior approach to the tibia or stand on the opposite side of the table
7	Rotation of the tibia is often increased in collateral ligament injuries and facilitates access to the posterior tibia

This study has several limitations. Although the short-term follow-up allows assessment of complications, long-term follow-up is necessary to understand the risk of increased posttraumatic osteoarthritis [19]. Because of the low-resource setting and difficulty contacting patients, we were unable to perform any instrumented ligamentous testing. But the follow-up was sufficient to assess perioperative complications and describe a safe technique to perform this procedure. Other limitations include the retrospective design, small number of patients, and lack of a standardized treatment protocol, all of which are common challenges in knee dislocation research.

Our preferred approach is an extended anteromedial incision for bicruciate injuries involving the medial side (KDIII-M). A lateral incision is included if the PLC structures are injured (KDIII-L) or when all ligaments are affected (KDIV). Alternatively, an anterior midline incision can be used to address all cruciate and collateral ligaments, thus avoiding multiple scars and potential wound healing problems; however, it requires extensive undermining of the skin to reach the collateral ligaments and tibial attachment of the PCL. Furthermore, accessing and protecting the peroneal nerve during PLC reconstruction can be challenging.

Other authors have described an open approach to the PCL tibial insertion with similar outcomes but this was usually done in a prone patient [7, 21–23]. The described approach allows maximum visualization of the PCL tibial footprint while protecting the neurovascular structures. Our technique can be performed in supine patients, enabling improved access to other injuries in patients with multiple traumas. Although most cruciate ligament reconstructions are performed arthroscopically [24], acceptable long-term follow-up can be achieved after open reconstruction [25]. Furthermore, a detailed summary of results of studies using open PCL [26] and ACL reconstructions [27] has been made in a meta-analysis and no difference in PROMs or complications was found when compared to arthroscopic surgery. Also, reported stiffness and HO after arthroscopic or closed treatment of knee dislocations are similar [19, 20].

Another important point to consider is that preoperative imaging is important to confirm lateral or medial laxity. This dictates the approach to the posterior part of the knee. MRI has high diagnostic accuracy in acute knee dislocations [28], but when MRI is not available, similar accuracy can be achieved with comparative clinician-assisted varus and valgus stress radiographs in 20° of flexion [29–31]. Here the tibiofemoral distance at the medial or lateral joint line is measured in millimetres and compared to the contralateral side. A similar accuracy has also been found with PCL stress views. These can be done with posteriorly directed force by the clinician, or in form of kneeling radiographs [32]. Here the line of the posterior tibial cortex is referenced to the most posterior point of the Blumensaat line and this distance is compared to the contralateral side. A further advantage of stress radiographs is their dynamic component of assessment and the possibility to grade the laxity based on the distance measured (Table 3). Although preoperative assessment prior to surgery is essential for adequate planning, stress radiographs done under anaesthesia and before the incision can therefore provide crucial information even if MRI is available.

**Table 3:** Evaluation of posterior, varus, and valgus knee instability using stress radiographs [3].

Kneeling stress	Injury	Grade	Varus stress	Injury	Valgus stress	Injury
≤7mm	Normal or partial tear	I	≤2.6mm	Normal or partial tear	≤3.1mm	Normal or partial tear
8–11mm	Complete PCL tear	II	2.7–3.9mm	Isolated LCL tear	3.2–9.7mm	Complete sMCL tear
≥12mm	Combined ligament injury	III	≥4mm	Complete PLC injury	≥9.8mm	Complete tear of all medial structures

LCL, lateral collateral ligament; PCL, posterior collateral ligament; PLC, posterolateral corner; PTT, posterior tibial translation; sMCL, superficial medial collateral ligament.

Overall, our findings support the concept that open approaches to knee dislocations can be a useful tool for cruciate ligament reconstruction in special circumstances. The most important future potential for it is the management of MLKIs in LRS.

## Supplementary Material

Supplementary material is available at https://www.sicot-j.org/10.1051/sicotj/2021016/olm *Video 1*. Posteromedial approach cadaver JSICOT. *Video 2*. Notch access JSICOT. *Video 3*. Posterolateral approach cadaver JSICOT. *Video 4*. Posteromedial approach surgery JSICOT.

## Conflict of interest

DW receives funding from Smith & Nephew and Axogen in the form of educational grants unrelated to the submission. MH receives funding from Smith & Nephew in the form of educational grants unrelated to the submission. ML, RvB, DR, and RS certified that they have no financial conflict of interest (e.g., consultancies, stock ownership, equity interest, patent/licensing arrangements, etc) in connection with this article.

## Authorship statement

All authors have contributed substantially to the conception of the work, the drafting of the manuscript and have approved the final version to be published and agree to be accountable for the work.
